# Hair as Suture and Ligature

**Published:** 1871-01

**Authors:** 


Hair as a Suture and Ligature.—Since 1868, Dr. Darby has experimented to a considerable extent upon this subject; he was led to do this in view of the embarrassment surgeons have at times felt in the want of a material for sutures and ligature which possessed all the requisites such material ought to have, of strength, flexibility, pliability, non-absorbing power, and absence of any tendency to irritate. Hair seems to possess these qualities in an eminent degree; his experience with it has demonstrated its title to a place among the valuable materials for this part of the surgeon's work.

He takes clean black horse-hair, using one, or several hairs twisted together, according to the strength required in each particular case. The common surgeon's knot secures amply the ligature, or suture, but care is necessary that the hair is not twisted in folds, as it is likely to break at such points, and that the nails are not used in drawing the knot, as the hair may be thus easily cut through. Before using the hair, he washes it in a solution of carbolic acid, three or four grains to the ounce, to destroy any noxious material that may be upon it.

He has used hair in amptutations, removal of tumors, incised wounds of the scalp, penis, scrotum, neck, trunk, eyelids; in mucous membranes of the eye, mouth, arms, rectum, and vagina, in all of which immediate union is desirable; and, in most of the operations, gives it preference over all other materials.

In wounds of the rectum and vagina it is inferior to metallic sutures, from the difficulty of tying in so narrow cavities; the twist with the metal is more easily made. When wounds of these cavities are more superficial, the hair is the preferable suture.

In wounds of tissues which are lax and delicate, such as the eyelids, scrotum, and penis, the hair is superior to the metal, for being unirritating it leaves no scar, its pliability allows of its accomodation to the folds of the tissue, and its elasticity facilities its easy withdrawal.

In operations upon the eye and its appendages, it is more advantageous than silk, flax, or cotton; any irritation to the parts that might accrue from the stiff ends of the hair is obviated by leaving the sutures long enough to be fixed to the cheek by adhesive plaster; or the same object may be gained by using human hair, or that from the ox's tail, which are so fine and soft that the ends may be cut short without fear or injury to the lids.

He has allowed sutures to remain in wounds from ten to twenty days without irritation.—*Richmond and Louisville Medical Journal.*

